# Limits Of Quantum Information In Weak Interaction Processes Of Hyperons

**DOI:** 10.1038/srep11591

**Published:** 2015-07-06

**Authors:** B. C. Hiesmayr

**Affiliations:** 1 University of Vienna, Faculty of Physics, Boltzmanngasse 5, 1090 Vienna, Austria

## Abstract

We analyze the achievable limits of the quantum information processing of the weak interaction revealed by hyperons with spin. We find that the weak decay process corresponds to an interferometric device with a fixed visibility and fixed phase difference for each hyperon. Nature chooses rather low visibilities expressing a preference to parity conserving or violating processes (except for the decay Σ^+^→ *p*π^0^). The decay process can be considered as an open quantum channel that carries the information of the hyperon spin to the angular distribution of the momentum of the daughter particles. We find a simple geometrical information theoretic interpretation of this process: two quantization axes are chosen spontaneously with probabilities 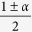 where *α* is proportional to the visibility times the real part of the phase shift. Differently stated, the weak interaction process corresponds to spin measurements with an imperfect Stern-Gerlach apparatus. Equipped with this information theoretic insight we show how entanglement can be measured in these systems and why Bell’s nonlocality (in contradiction to common misconception in literature) cannot be revealed in hyperon decays. Last but not least we study under which circumstances contextuality can be revealed.

Weak interactions are one out of the four fundamental interactions that we think that rules our universe. The weak interaction is the only interaction that breaks the parity symmetry and the combined charge-conjugation–parity (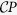) symmetry. Recently, it has been shown that for K-mesons^1^, which are spinless particles decaying via the weak interaction, Bell’s theorem can be related to the violation of the 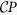 symmetry. Herewith one of the most counterintuitive property of quantum mechanics relates itself to the unsolved problem of the matter-antimatter asymmetry in our universe. This paper focus on particles having half-integer spin and decaying via the weak interaction, i.e. hyperons that are baryons containing in addition to up or down quarks also one or more strange quarks. Hyperon decays violate the parity (*P*) symmetry. The Standard Model of elementary particles predicts also tiny contribution of 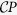 violating processes, however, no violation of the 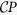 symmetry has been up to now experimentally found.

We develop an information theoretic description of the weak decay process of hyperons which also puts the new expected data (e.g. by the PANDA experiment^2^ at FAIR in Germany and BES-III^3^ at BEPC II in China) into a unified picture, connects it to quantum information theory and clears some misunderstandings existing in the literature. In particular, we find that the *P* violating and non-violating amplitudes correspond to the two arms of an asymmetric interferometer allowing for a quantified discussion of Bohr’s complementarity relation. We can further show that the decay process is a noisy quantum channel or differently stated, the spin of the decaying particle is only imperfectly measured by the decay process. Equipped with this quantum information theoretic insight we proceed to the two-particle case where experimental data suggests that there is entanglement in the system. We introduce an optimal observable that witnesses the entanglement in the spin degrees of freedom. Last but not least we discuss whether a test versus local realistic theories via Bell inequalities is possible and whether the contextuality property of the quantum theory can be revealed in hyperon-antihyperon systems.

## Results

### Information theoretic content of an interfering and decaying system

Let us start by assuming that the initial state of a decaying hyperon is in a separable state between momentum and spin degrees of freedoms *ρ*_hyperon_ = *ρ*_*mom*_ ⊗ *ρ*_*spin*_. Typically one is interested in the angular distribution of the decay products (*θ*, *ϕ*) depending on the polarization state of the hyperon particle, i.e. a projection onto the above state
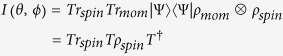
where *T* is usually dubbed decay or transition matrix. Given a specific symmetry, e.g., the parity symmetry, one may have two different processes: one conserving, the other one violating this symmetry in the decay process. Let us label these two different possibilities with *a* and *b*, then we have
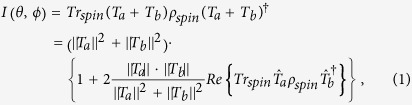
where we considered for the last equation the normalized quantities 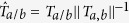. The coefficient in front of the interference term can be considered as the visibility 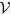, the interference contrast of the two interfering amplitudes. A predictability 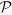 can be derived by computing the difference between the probability that a particle decays via the process *a* and *b*


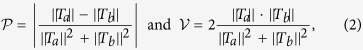


and, obviously, we have

**Figure 1:**
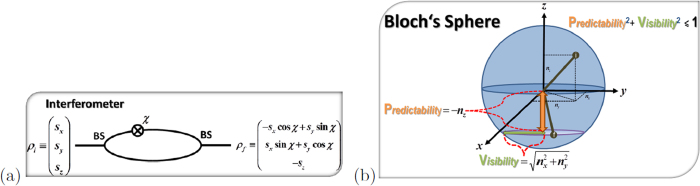
(**a**) depicts an interferometer for a spin-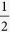 particle with spin oriented along 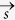 propagating through two beam-splitters (BS) and picking up a relative phase *χ*. In (**b**) the Bloch’s sphere is depicted and shows how a given interferometric setup changes the initial spin. Depending on the initial spin the interferometer reveals “*wave-particle*” property, quantified by the visibility, or “*which-way*” information, quantified by the predictability. Both quantities add up to one for pure spin states.

*V*^2^ + *P*^2^ = 1,

which is a quantitative rephrase of Bohr’s complementarity relation or the closely related concept of duality in interferometric devices (for an illustration see Fig. 1). Predictability and visibility is a pair of complementary properties, so the better we know one of them, the less we can determine the other one. One may relate predictability to the “*particle-like property*”, i.e. a “*which way*”–information, and visibility to the “*wave-like property*”, i.e. the interference contrast or sharpness. The first step in bringing the qualitative statement “*the observation of an interference pattern and the acquisition of which-way information are mutually exclusive*” into a quantitative one was taken by Greenberger and Yasin^4^ and refined by Englert^5^. The authors of Ref. 6 investigated physical situations for which the complementary expressions depend only linearly on some variable *y*. This included interference patterns of various types of double slit experiments (*y* is linked to position), but also oscillations due to particle mixing (*y* is linked to time), e.g. by the neutral K-meson system, and also Mott scattering experiments of identical particles or nuclei (*y* is linked to a scattering angle). For the K-meson system the effect of the 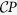 symmetry violation was investigated^7^ showing that it shifts obtainable information about our reality to different aspects, without violating the complementarity principle, i.e. from predictability 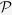 to visibility 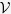 and vice versa. All these two-state systems belonging to distinct fields of physics can then be treated via the generalized complementarity relation in a unified way. In Ref. 8 the authors investigated how the un-stability due to a decay within the interferometer reduces the visibility.

### An open quantum formalism for decaying hyperons

Quite generally, we can rewrite the dynamics in the spin space by switching to the formalism of open quantum systems that allows a fast computation and transparent interpretation. In particular, we can always find two hermitian Kraus operators *K*_±_ being the sum of the real and imaginary part of the decay matrix such that the following equation holds under the trace operation (explicit examples are given later)
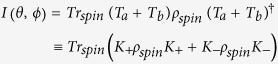
where the Kraus operators have the conceptually simple form (*ω*_±_ > 0)


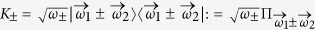


with *ω*_+_ + *ω*_−_ = 1. The two Blochvectors have to be orthogonal, 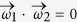, since the transition is completely positive and are chosen such that they have maximal length 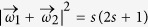 (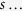 spin number). A Blochvector expansion of a density matrix is generally given by 
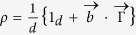
 where *d* is the dimension of the system^9^. Since we are dealing with spin-degrees of freedom we have *d* = 2*s* + 1 and we can choose as a set of orthonormal basis the generalized Hermitian and traceless Gell-Mann matrices 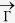 (for 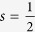 they correspond to the Pauli matrices). Note that one can assign to any density matrix a (real) Bloch vector but not all real vectors correspond to a density matrix except for the lowest possible dimension 2.

**Figure 2:**
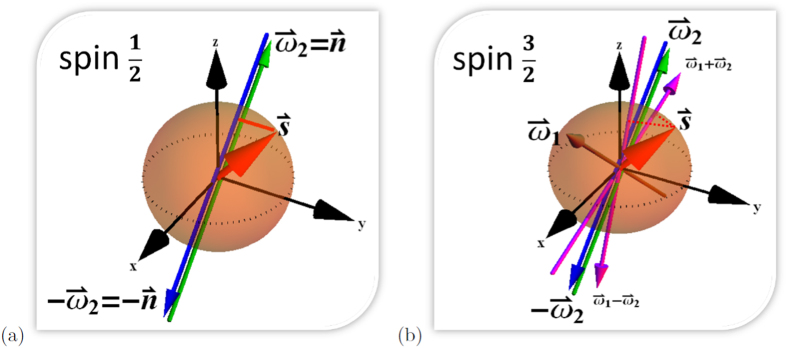
(**a**) shows the three dimensional Bloch’s sphere for spin-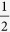 particles and (**b**) the eight dimensional Bloch’s sphere for spin-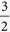 particles. Weak interaction depicts two quantization directions 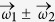 along which the initial spin of the hyperon is projected. For spin-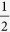 particles 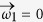 and with probability *ω*_±_ the hyperon spin is projected onto 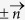 where 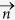 corresponds to the momentum direction of the daughter particle. In case of spin-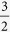 particles the real and imaginary parts of the transition amplitude differ resulting in a contribution to the quantization directions that is independent of difference of the two probabilities *ω*_±_. Hence the hyperon spin is projected onto two slightly tilted quantization directions 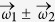.

Given this structure we can reinterpret the weak decay process as an incomplete spin measurement of the decaying particle
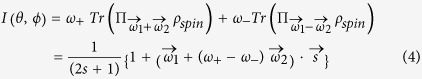
where *s* is the Bloch vector representation of *ρ*_*spin*_, i.e. 
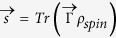
. With probability *ω*_+_ the spin state of the hyperon is projected onto direction 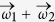 or with the remaining probability *ω*_−_ the initial spin state is measured along the direction 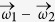. Thus the weak process can be associated to a spin measurement with an imperfect Stern-Gerlach apparatus (switching with probability *ω*_±_ the magnetic field) which is geometrically depicted in Fig. 2. The imperfection has two causes: Firstly, the difference (*ω*_+_ − *ω*_−_) equals an asymmetry (denoted in the following by *α*) which corresponds to the characteristics of the interferometer. Secondly, the two directions 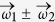 are characteristical for the weak decay. Explicit examples are given below.

Let us remark here that for a spin-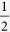 particle the decay dynamics simplify considerably since the real and imaginary part of the transition amplitude *T*_*a*_ + *T*_*b*_ are equal, leading to 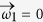. Thus we can say that Nature chooses between two opposite directions 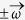 albeit between two different handed coordinate systems or chirality. Of course, we could also reinterpret equation (4) if and only if the initial spin state is pure stating that the spin direction is projected onto the mixed momentum state 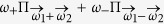. This interpretation does not hold if there is an initial correlation between spin and momentum degrees of freedom, e.g. for decay cascades (Example III).

#### Example I: A spin- 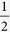 particle propagating through an interferometer

Let us consider a spin-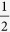 particle that passes a beam-splitter (BS). The interaction can be reasonably well described by the unitary operator 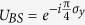. Then the particle picks up usually a phase shift 
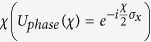
 and passes again a beam-splitter. A pure initial spin state 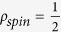

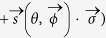
 is then changed by a total unitary operation 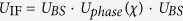 into a final state (see also Fig. 1)


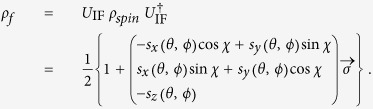


A measurement in *x*- or *y*-direction reveals the interference
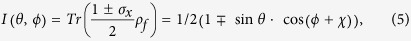
and therefore the visibility is in this case 
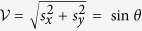
. Whereas a measurement in *z*-direction reveals the probability to propagate via the upper or lower path of the interferometer
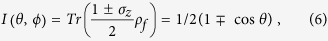
and therefore the “*which way*” information is quantified by the predictability 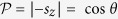, i.e. the initial spin contribution in *z*-direction. Thus 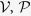 can be chosen via the preparation of the initial spin (given the interferometric setup).

In the following we go one step further and assume that one has an interferometric device including a measurement along *x*- or *y*-direction, albeit revealing the “*wave particle*” information of the spin, however, we now change the probability of the beam-splitter to a 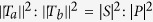 one and find (see Eq. (1))
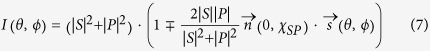
by choosing the following *T* matrices


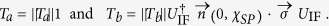


With Eq. (2) we obtain a quantified quantum information theoretic interpretation of Bohr’s complementarity relation of the interferometer in terms of 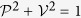, i.e. how much interference contrast corresponds to a certain interferometric choice. Note the conceptual difference to the previous scenario where 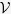 referred to the chosen initial spin. We are therefore not limited to spin-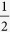 decays since we are interested in the two amplitude process of the weak interaction. Let us apply now this view to hyperon decays.

#### Example II: Spin- 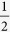 hyperon nonleptonic decays

The conservation of the total spin implies that the final states can have two different angular momentum eigenstates. We denote with *S*/*P* the amplitude of the parity violating/conservating process (corresponding to the *S*, *P* wave with angular momentum *l* = 0, 1), then the decay matrices are 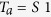 and 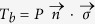 computed via the corresponding spherical harmonics and Clebsch-Gordan coefficients^10^. The angular distribution of the normalized momentum 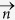 of the daughter particle is given by Eq.(4) with 
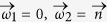
 and *ω*_+_ − *ω*_−_ = *α*. Depending on the production process symmetries on 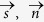 may be superimposed. In the standard phenomenology of hyperon decays the following decay parameters are introduced^11^
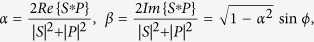

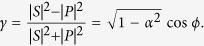
and the measured values of the parameters *α*, *ϕ* are given in Ref. 12. We can connect these parameters to our information theoretic quantities 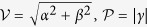 and 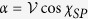. Time reversal symmetry requires, in the absence of final-state interactions, that the *S*- and *P*-wave amplitudes should be relatively real, hence *ϕ*  ≈ 0, which means that the asymmetry parameter *α* approximately equals the visibility 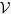 of the *S* and *P* wave.

In the table 1 we list all hyperon decays for which *α* and the phase *ϕ* have been measured. Let us remark here that if *ϕ* is not measured we cannot say anything about the visibility since it depends strongly on *ϕ* except for *α* close to 1. We observe that the phase shifts *χ*_*SP*_, which reduce the total visibility of the decay, are rather small. For some decays, where the phase shifts are known with limited accuracy, a larger values of the phase shifts (up to ≈ 0.5π) are still possible. The predictability of all weak decay processes is rather large except for the Σ^+^ particle. This particle has –in contrary to the other hyperons– two decay channels with nearly equal branching ratios. Due to the rule for the hyper charge 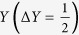 and the spurious-kaon rule^11^ identical *α*’s and *ϕ*’s are expected for the following two pairs of decays: Λ → π^−^*p*; π^0^*n* and Ξ^−,0^ → Λπ^−,0^. That is significantly different to the case of the Σ^±^ decays where the visibility is close to zero. This implies that one of these decays must be mainly a *S*-wave and the other one mainly a *P*-wave. This means also that the imperfection of the spin measurement is low in strong contrast to Ξ^−^ decays where the probability of both directions is close to 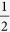.

In summary, the weak decay process of hyperons with spin can be viewed as an interferometer device with a fixed phase and a beam splitter with a fixed splitting. This corresponds to fixed visibilities and predictabilities independently of the initial spin of the decaying hyperon.

#### Example III: Cascade of hyperon decays

Considering subsequent decays of hyperons into hyperons and finally into non-strange particles we can apply the transition matrices *T*_*μ*_, *T*_*ν*_ in a straightforward way by
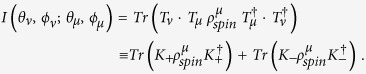


The last equation holds since we have again a complete positive evolution. Let us remark here that these Kraus operators *K*_±_ are not a product of the Kraus operators of each decay. The reason is that after the first decay we have correlations between the spin and momentum degrees of freedom, i.e. the crucial initial condition *ρ*_*mom*_ ⊗ *ρ*_*spin*_ does not hold. Contrary to the dynamics of closed systems open quantum systems have no continuity of time and therefore it is not always possible to formulate the general dynamics by means of differential equations generating contractive families. On the contrary cascades allow to reveal also more information on the *S*-wave and *P*-wave coefficients, i.e. after a straightforward computation we find for the decay of a hyperon *μ* (spin-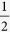) into a hyperon *ν* (spin-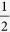) and finally into a baryon (spin-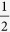) the following Kraus coefficients 
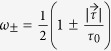
 and 
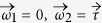
 with
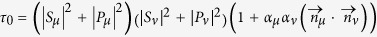

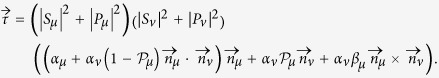


The angle between the momenta of the two hyperons 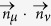
is fixed by the request to orientate the two reference systems with respect to the initial hyperon spin. We obtain a conceptually simple interpretation of the decay cascade as in Example II: the two quantization directions are 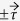
and the momentum direction of the baryon is measured with probability 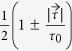 along them. Notable that 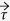 has a contribution 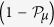 along the direction of the momentum of the daughter hyperon 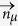, whereas the direction of the momentum of the baryon 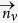 is multiplied by the predictability . For example the predictabilities of the two decay cascades Ξ^−,0^ → Λπ^−,0^ → *p*π^−,0^π^−^,*n*π^−,0^π^0^ are large 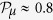 and therefore the quantization axis becomes to a good approximation 
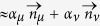
.

### Entanglement

Entangled 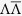 pairs can be produced for example via proton-antiproton annihilation and the full spin structure can be experimentally determined^13^. In this section we analyse the entanglement properties and discuss whether Bell’s nonlocality or contextuality can be revealed in such experiments. Let us assume that (i) there is no initial correlation between the momentum degrees of freedom and the spin degrees of freedom and (ii) there is no entanglement between the momentum degrees of freedom. Our formalism extends straightforwardly to the two-particle case by taking the tensor product of the Kraus operators. Experiments^13^,^14^,^15^ suggest that the initial spin state is a maximally entangled Bell state (except for backward scattering angles). Therefore without loss of generality we can choose the antisymmetric Bell state 
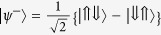
 and the angular momentum distribution becomes
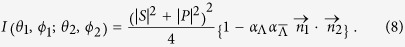


Since 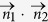 is multiplied by a constant 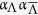 Törnquist^16^ concluded that Λ decays “*as if it had a polarization α*_Λ_ *tagged in the direction of the* π^+^*(coming from the* 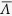
*) and vice versa*”. The knowledge of how one of the Λ'*s* decayed –or shall decay (since time ordering is not relevant)– reveals the polarization of the second Λ. He concludes that this is the well-known Einstein-Podolsky-Rosen scenario.

**Figure 3:**
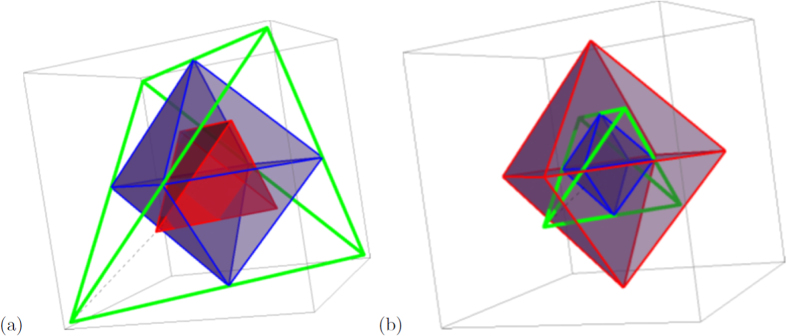
The big (green) tetrahedron corresponds to the geometrical illustration of all local maximally mixed bipartite qubit states which can be represented by three real numbers. The four corners represent the four maximally entangled Bell states which are the only pure states in the picture. Separable states are those inside the (blue) double pyramid where the surfaces correspond to optimal entanglement witnesses. For the 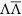 system we can interpret that **(a**) the Hilbert space shrinks with the factor 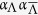 visualized by the small (red) tetrahedron. Since the corners are still outside of the optimal entanglement witnesses (double pyramid), entanglement can be detected via the weak decaying process. Or equivalently, as illustrated in (**b**) we could also say that weak interactions correspond to imperfect spin measurements blowing up the optimal entanglement witnesses, huge (red) double pyramid.

In general entanglement is detected by a certain observable that can witness the entanglement content, i.e. a Hermitian operator 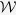 for which holds 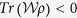 for at least one state *ρ* and 
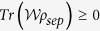
 for all separable states *ρ*_*sep*_. For the antisymmetric Bell state such an optical entanglement witness is given by 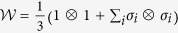 (any other witness can be obtained by local unitary transformations). Since the weak interaction only allows for an imperfect spin measurement we have to multiply the spin part by 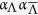. Thus 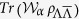 results in
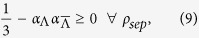
which is clearly violated since 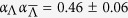
12. Therefore, the measurement of the correlation functions 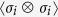 in *x*, *x* and *y*, *y* and *z*, *z* directions of the Λ and 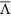 reveals entanglement. Generally, we can say that the asymmetries lead to imperfect spin measurements which shrink the observable space. Equivalently, we can say that the given interferometric device leads to a shrinking of the Hilbert space of the accessible spin states. In Fig. 3 we have visualized the geometry of the Hilbert space for all locally mixed states of bipartite qubits forming a magic simplex^17^,^18^,^19^. Locally mixed states are those for which any partial trace reduces to the maximally mixed state. This set of states can be described by three real numbers. Positivity requires that these three numbers form a tetrahedron with the four maximally entangled Bell states in the corners. The separability condition corresponds to a double pyramid. The surfaces of the pyramid correspond to the optimal entanglement witnesses. As shown in Fig. 3 the factor 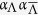 (a) shrinks the total state space (smaller red tetrahedron) or (b) blows up the optimal entanglement witnesses. Since 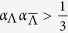 one can distinguish between entangled and separable states directly, i.e. without the additional information coming from the two interferometer devices.

### Testing for local realism or contextuality

The next question is whether the entanglement observed in this system manifests itself in the most counterintuitive properties of quantum theory, i.e. Bell’s nonlocality or contextuality. The notion of contextuality, introduced by John Bell^20^ and by Kochen and Specker^21^, can be explained as follows. Suppose that a measurement *A* can be jointly performed with either measurement *B* or *C*, i.e. without disturbing the measurement *A*. Measurements *B* and *C* are said to provide a context for the measurement *A*. The measurement *A* is contextual if its outcome depends on whether it was performed together with *B* or with *C*. Therefore, the essence of contextuality is the lack of possibility to assign an outcome to A prior to its measurement and independently of the context in which it was performed. The seemingly different Bell theorem is in fact a special instance of the Kochen-Specker theorem where contexts naturally arise from the spatial separation of measurements. The usual toolboxes for revealing nonlocality and contextuality are state in-dependent or dependent inequalities of probabilities. In the following we analyze these inequalities for the hyperon-antihyperon system.

We considered Bell inequalities for qubits with two, three or four different choices of observables for both particles, i.e. the following expressions
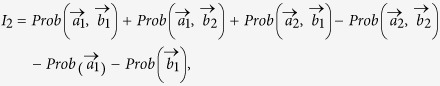

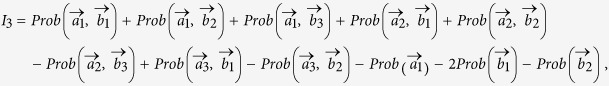

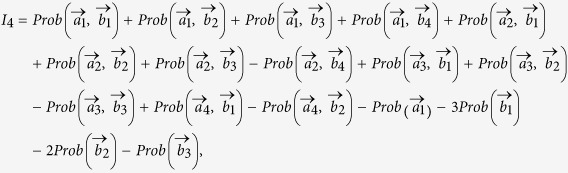
where 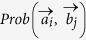 is the probability that the first particle is measured along 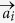 giving e.g. the plus result and the second particle is measured along the direction 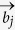 and gives e.g. the plus result. For any local realistic theories *I*_*i*_  ≤ 0 has to hold. We find that the strongest constraint is found for the famous CHSH-Bell inequality^22^ (*I*_2_), i.e.
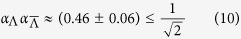
that has to hold for all local realistic theories. Clearly, this reveals no violation for the 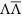 system. However, we have to remark that for testing theories based on local hidden parameters versus the predictions of quantum mechanics there are two important requirements: Firstly, one is not allowed to refer to results that are deduced from quantum mechanical considerations. In our case this means that we cannot re-normalize to the asymmetry parameters 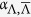 since they are obtained from quantum mechanical properties. Secondly, any conclusive Bell’s test requires that an active control of the experimenter about measurement setting is given, i.e. a choice of freedom which property of the state will be measured. Otherwise it is straightforward to construct a local realistic hidden variable theory to explain the data. This active control is clearly not available for weakly decaying hyperons since the quantization axes are spontaneously chosen. Some authors^23^ argued that one can circumvent the requirement since an observer can choose a coordinate system at will at each side. We disagree since one needs always one common reference system to describe the two-particle decay. Note that if there would be entanglement in the momentum state then this entanglement can (in some cases) be transferred to the spin states for a relativistically boosted observer^24^. However, in this case also the operators corresponding to the measurements are boosted in exactly such a way that the expectation value is independent of the considered reference frame in agreement with special relativity. Hence a violation of a Bell inequality found in one reference system is also violated in another one. In summary, in contrary to the weakly decaying spinless K-meson system^1^ the active measurement procedure for hyperons decays are not available and therefore no contradiction to the premisses of local realism based on Bell inequalities can be derived.

Contextuality tests usually do not suffer from these requirements. Due to the imperfect spin measurements we expect for the hyperons a decrease of probabilities and therefore a dependence of the violation on the asymmetry term. The tests need the property of joint measurements that is available for the hyperon system since we have the tensor product between the observables. If one follows the interpretation that the accessible state space is shrunken (that holds true only if there is no initial correlation between the momentum degrees of freedom and the spin degrees of freedom), then all state independent proofs such as the Mermin-Peres square^25^,^26^,^27^,^28^ hold also for the 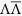 system and contextuality is revealed. Applying the other interpretation that holds also in the general case (see example III) the observables have to be multiplied by *α*_Λ_ or 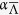, respectively, and the Mermin-Peres inequality leads to
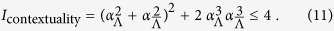
which is not violated. Assuming 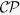 conservation contextuality would be revealed if *α* > 0.88 and hence greater than the bound from the Bell inequality *α* > 0.84.

## Discussions

In this contribution we introduce an information theoretic approach to hyperons decaying via the weak interaction. The parity violating and non-violating processes can be considered as two different paths in an interferometer. We find that weak interaction chooses for each hyperon an interferometric device with a fixed visibility, i.e. interference contrast, and a fixed phase shift. The visibility results from a non-symmetric beam-splitter. Based on Bohr’s complementarity relation we can derive the related predictability, quantifying the “*particle-like*” property, which turns out to be in general high (except for one decay mode of the Σ^+^ hyperon). Thus weak interaction distinguishes strongly between processes conserving and not conserving the parity symmetry.

Applying the open quantum formalism we find a simple and transparent method to describe weakly decaying hyperons. We find that the decay via the weak interaction corresponds to an imperfect spin measurement, where right and left-handed coordinate systems mix.

Equipped with this quantum information theoretic knowledge we proceeded to two-particle system, e.g. the 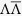 system. Entanglement can be proven experimentally although the imperfection in the spin measurements. We show further that it is impossible to find a conclusive contradiction between quantum theory and local realistic theories via Bell inequalities since weak decays offer no active control over the quantization directions. Last but not least we investigate contextuality in the 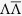 system. We find that it cannot be revealed by the Mermin-Peres square if one assumes that the violation of the parity symmetry shrinks the accessible observable space. The second possible interpretation —violation of the parity symmetry corresponds to the shrinking of accessible state space— would reveal the contextual nature of quantum theory, however, this interpretation does not hold if there are correlations between the spin and momentum degrees of freedom. In opposition to the weakly decaying K-meson system the observation of the violation of the 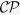 symmetry would not considerably alter the results.

We believe that the presented information theoretic analyzes of hyperon decays and entanglement helps to bring the data from the upcoming experiments in a unified picture and contributes to the understanding of weak interactions and the importance of the parity violation for the development of our universe.

## Additional Information

**How to cite this article**: Hiesmayr, B. C. Limits Of Quantum Information In Weak Interaction Processes Of Hyperons. *Sci. Rep.* **5**, 11591; doi: 10.1038/srep11591 (2015).

## Figures and Tables

**Table 1:** The information theoretic content of the weak decay processes violating the strangeness number.

**hyperon (quarks)**	**decay channel (frequency)**	**phase shift *χ*_*SP*_ in[π]**	**visibility** 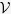	**predictability** 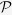
Λ(*uds*)	*p*π^−^ (63.9%)	−(0.043 ± 0.023)	0.648 ± 0.014	0.762 ± 0.012
	*n*π^0^ (35.8%)	−(0.042 ± 0.023)	0.656 ± 0.040^*^	0.755 ± 0.034^*^
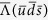	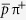 (63.9%)	0.036 ± 0.021	0.714 ± 0.079^*^	0.700 ± 0.080^*^
Σ^−^(*dds*)	*n*π^−^ (99.8%)	−(0.38 ± 0.16)	0.19 ± 0.24	0.98 ± 0.05
Σ^+^(*uus*)	*p*π^0^ (51.6%)	−(0.038±0.035)	0.976 ± 0.016	0.161 ± 0.097
	*n*π^+^(48.3%)	0.41 ± 0.13	0.24 ± 0.33	0.972 ± 0.078
Ξ^0^(*uss*)	Λπ^0^ (99.5%)	0.214 ± 0.085	0.53 ± 0.11	0.85 ± 0.07
Ξ^−^(*dss*)	Λπ^−^ (99.8%)	0.0226 ± 0.0086	0.459 ± 0.012	0.8884 ± 0.0062

The values and errors are taken from the particle data book^15^. The asterisk ^*^ denotes that the phase *ϕ* was not independently measured but deduced via conservation laws from the other decay modes. 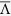 also decays into *n*π^0^, however, it has not yet been measured. The only non-resonant hyperons with spin-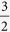 are the Ω^±^ particles, the measured asymmetry are close to zero (*α*_Ω_ = 0.0180 ± 0.0024) and no *ϕ* information is available.
